# Mixed β-γ-Cyclodextrin Branched Polymer with Multiple Photo-Chemotherapeutic Cargos

**DOI:** 10.1021/acsapm.3c01157

**Published:** 2023-08-31

**Authors:** Francesca Laneri, Mimimorena Seggio, Cristina Parisi, Szabolcs Béni, Aurore Fraix, Milo Malanga, Salvatore Sortino

**Affiliations:** † PhotoChemLab, Department of Drug and Health Sciences, University of Catania, I-95125 Catania, Italy; ‡ Department of Pharmacognosy, Semmelweis University, I-1085 Budapest, Hungary; § CycloLab, Cyclodextrin R&D Ltd., I-1097 Budapest, Hungary

**Keywords:** light, cyclodextrin, nitric oxide, supramolecular assembly, multimodal therapy

## Abstract

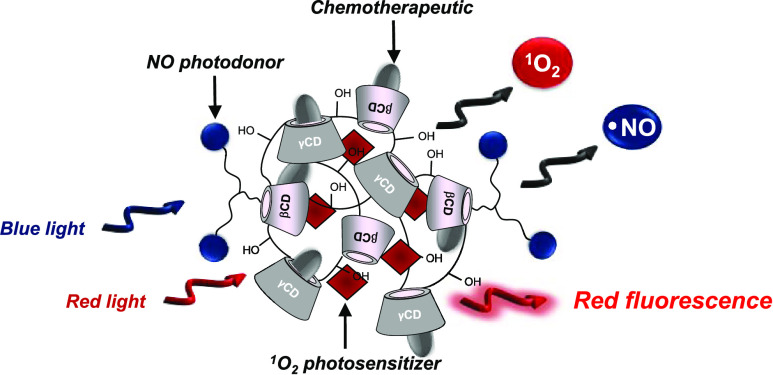

The achievement of biocompatible platforms for multimodal therapies is one of the major challenges in the burgeoning field of nanomedicine. Here, we report on a mixed β- and γ-cyclodextrin-based branched polymeric material (**βγCD-NOPD**) covalently integrating a nitric oxide (NO) photodonor (NOPD) within its macromolecular scaffold, and its supramolecular ensemble with a singlet oxygen (^1^O_2_) photosensitizer (PS) Zn(II) phthalocyanine (**ZnPc**) and the chemodrug Lenvatinib (**LVB**). This polymer is highly water-soluble and generates NO under visible blue light stimuli with an efficiency of more than 1 order of magnitude higher than that of the single NOPD. The PS, which in an aqueous solution is aggregated and non-photoresponsive, can be entangled in the polymeric network as a photoresponsive monomeric species. In addition, the poorly water-soluble **LVB** can be co-encapsulated within the polymeric host, which increases the drug solubility by more than 30-fold compared to the free drug and more than 2-fold compared with a similar branched polymer containing only βCD units. The supramolecular nanoensemble, *ca.* 15 nm in diameter, retains well the photochemical properties of both the NOPD and PS, which can operate in parallel under light stimuli of different energies. Irradiation with blue and red light results in the photogeneration of NO and ^1^O_2_ associated with red fluorescence emission, without inducing any photodegradation of **LVB**. This result is not trivial and is due to the absence of significant, mutual interactions between the NOPD, the PS and **LVB** both in the ground and excited states, despite these components are confined in the same host. The proposed polymeric nanoplatform may represent a potential trimodal nanomedicine for biomedical research studies, since it combines the double photodynamic action of NO and ^1^O_2_, two species that do not suffer multidrug resistance, with the therapeutic activity of a conventional chemodrug.

## 1 Introduction

Multimodal therapies to fight cancer diseases exploit different treatment modalities with the goal of improving the therapeutic outcome through synergistic/additive actions while minimizing side effects.^1,2^ In this regard, a combination of conventional chemotherapeutics with unconventional light-generated therapeutics is a very appealing approach that has been receiving growing attention, especially over the last decade.^3,4^ Light is an ideal tool for the introduction of a cytotoxic species in a bioenvironment with very precise control of site and dosage through the straightforward regulation of the irradiated area and irradiation time.^5,6^

Among light-stimulated unconventional approaches to fight cancers, photodynamic therapy (PDT) represents the most promising and it also finds application in clinics.^7,8^ PDT is mainly based on the cytotoxic action of the highly reactive singlet oxygen (^1^O_2_).^9^ This species is much more oxidant than molecular oxygen and is generated in a catalytic fashion by collisional energy transfer between the excited triplet state of a photosensitizer (PS) and the molecular oxygen.^10^

In recent years, photodynamic treatments exploiting nitric oxide (NO) as unconventional therapeutics, namely, NO-PDT, have come overwhelmingly into the limelight and hold very promising features in cancer, although still confined to the research area.^11^ In addition to being a key bioregulator of a broad array of physiological processes,^12^ NO can act as an efficient antitumoral agent if produced at a suitable dosage. Micromolar concentrations of this diatomic free radical induce cell toxicity^13^ and inhibit the efflux pumps mainly responsible for multidrug resistance (MDR) in cancer cells.^14^ These properties have made the combination of NO-PDT with chemotherapeutics a very appealing approach to amplify the therapeutic effects of chemodrugs and reduce their dosage.^15−19^ However, it is also known that doses of NO produced in the nanomolar range can induce tumor proliferation.^20^ Due to these opposite effects, NO-PDT is based on light-activatable NO precursors, namely, NO photodonors (NOPDs) that allow the accurate regulation of the NO concentration by the appropriate tuning of the light intensity and irradiation time.^21−24^ Different from ^1^O_2_, NO is not generated through a catalytic mechanism. In this case, the excitation light is exploited to break a chemical bond, uncaging NO from a covalent molecular scaffold of the NOPD leading, of course, to the consumption of this latter.^21−24^ Therefore, the reservoir of NO is strictly dictated by the initial concentration of NOPDs.

Note that ^1^O_2_ and NO are multitarget “bullets” that do not suffer MDR phenomena and due to their short lifetimes (few μs and few s, respectively) confine their reactivity to short distances from their generation site (0.02–200 μm), minimizing systemic toxicity drawbacks typical of many chemotherapeutic drugs. Finally, NO photorelease does not depend on the presence of molecular oxygen, making NO-PDT complementary to PDT under hypoxia conditions, which are typical for some tumors.

On these grounds, the achievement of multifunctional supramolecular assemblies able to photogenerate ^1^O_2_ and NO and simultaneously incorporate conventional chemotherapeutics is a very challenging objective to pursue. In this regard, the significant breakthroughs in nanomedicine offer an unprecedented opportunity to design and fabricate polymeric nanoplatforms able to entrap multiple therapeutic/phototherapeutic agents in a single nanocarrier.^25,26^ This permits, in principle, the controlled delivery of several therapeutics in the same bioenvironment, encouraging their action on either a single oncogenic pathway through different mechanisms or across parallel pathways.

Branched polymers^27^ are impetuously emerging in view of their unique topological structures and physicochemical properties such as three-dimensional globular structure, small hydrodynamic radius, improved multifunctionality, and excellent encapsulation capabilities and water solubility, as recently described by Vicent and co-workers in a recent review paper.^28^ Among the rich variety of branched polymers, those based on cyclodextrins (CDs) are receiving growing interest as suitable macromolecular scaffolds for drug delivery.^29−31^ CDs are cyclic oligosaccharides based on 6 (αCD), 7 (βCD), or 8 (γCD) glucopyranose units, well known for their complexation, stabilization, and solubilization capabilities of a wide range of guest compounds.^32^ The most extensively used cross-linking reagent for the achievement of branched CD polymers is epichlorohydrin.^33^ βCD branched polymers have proven to be tolerated both *in vitro* and *in vivo*.^29−31^ We have demonstrated their suitability as host scaffolds for multimodal therapies with supramolecular systems combining PDT and NO-PDT^34−37^ as well as NO-PDT and chemotherapy.^18,19^ Recently, an interesting class of CD-based branched polymers containing both β and γCD units in the same macromolecular scaffold and obtained by green synthetic protocols has been reported.^38^ These mixed polymeric hosts offer a large number of binding sites with differentiated sizes and hydrophobic/hydrophilic features if compared with the analogues containing a single type of CD, permitting an easier allocation of multiple guests in different compartments.^39^ In the case of photoactivatable systems, this peculiarity assumes particular relevance because, in principle, it better avoids mutual guest interactions in the ground and excited states, which otherwise would modify the response to light in efficiency, nature, or both.

**Scheme 1:**
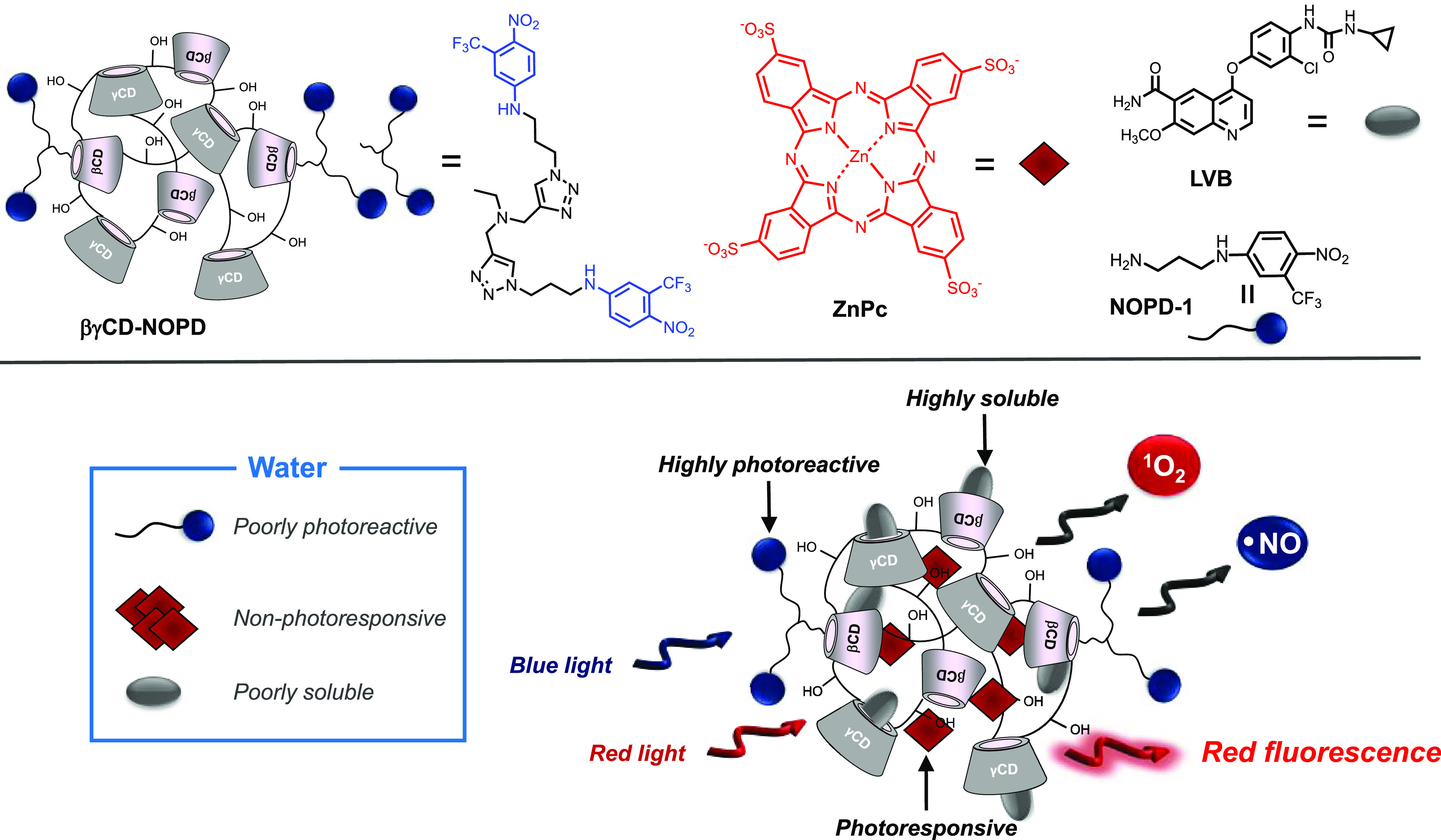
Top: Molecular Structures of the Mixed-Branched Polymer **βγCD-NOPD**, the PS **ZnPc**, the Chemodrug **LVB**, and the Model **NOPD-1**. Bottom: Schematic View of the Trimodal Supramolecular Assembly

With the aim to move toward trimodal photo-chemotherapeutic platforms, we report herein a mixed β- and γCD-based branched polymeric material **βγCD-NOPD**, which covalently integrates a nitroaniline derivative as NOPD within its macromolecular skeleton (Scheme 1). We demonstrate that this polymer (i) releases NO under blue light with efficiency higher than that of the individual NOPD unit, (ii) encapsulates the Zn(II) phthalocyanine tetrasulfonate **ZnPc**, a PS for PDT totally aggregated and non-photoresponsive in water, mainly under its photoresponsive monomeric form, (iii) co-encapsulates the poorly water-soluble chemotherapeutic Lenvatinib (**LVB**), increasing its solubility of ∼35-fold compared with the free drug, and (iv) preserves well the individual properties of all photo-chemotherapeutic components despite their confinement within the same host (Scheme 1). To our knowledge, no similar systems are known to date.

## 2 Results and Discussion

### 2.1 βγCD-NOPD and Its Binary Assemblies with LVB and ZnPc

**Scheme 2:**
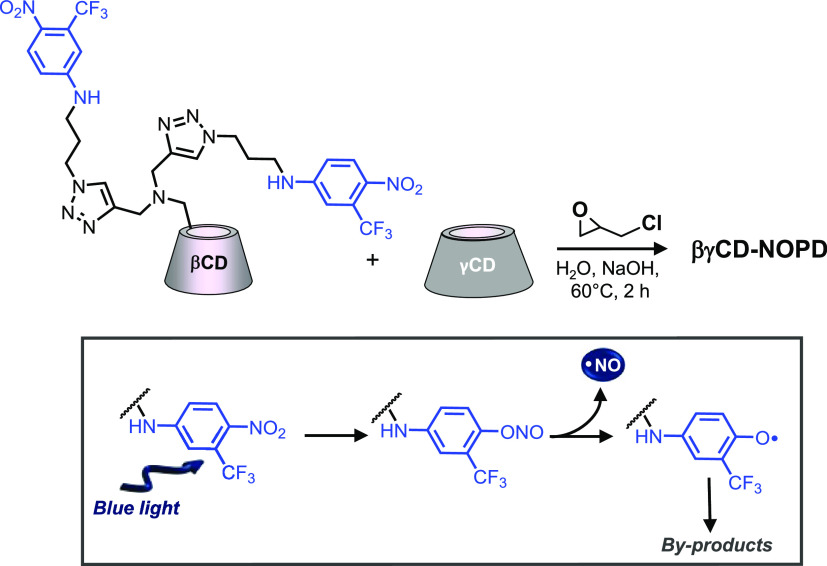
One-Step Copolymerization of the βCD Monomer Functionalized with Two NOPD Units and the Native γCD Using Epichlorohydrin as a Cross-Linker to Give the Mixed-Branched Polymer **βγCD-NOPD** The inset shows a sketch of the mechanism for the photochemical NO release from the NOPD unit.

**βγCD-NOPD** was synthesized in one step from the copolymerization of a functionalized βCD monomer and native γCD using epichlorohydrin as a cross-linker (Scheme 2) and described in detail in the Supporting Information. The functionalized βCD monomer integrates two units of a 4-nitro-3-(trifluoromethyl)amino-derivative, an NOPD developed in our group,^40^ which have been covalently linked to the lower rim of the macrocycle through click-chemistry starting from suitable precursors (see the Supporting Information). Analogously to other sterically hindered *ortho*-substituted nitroderivatives,^41^ this NOPD uncages NO under blue light irradiation through a mechanistic pathway involving a nitro-to-nitrite rearrangement, the homolytic rupture of the O–NO bond and the formation of a phenoxy radical (inset of Scheme 2).

**Figure 1:**
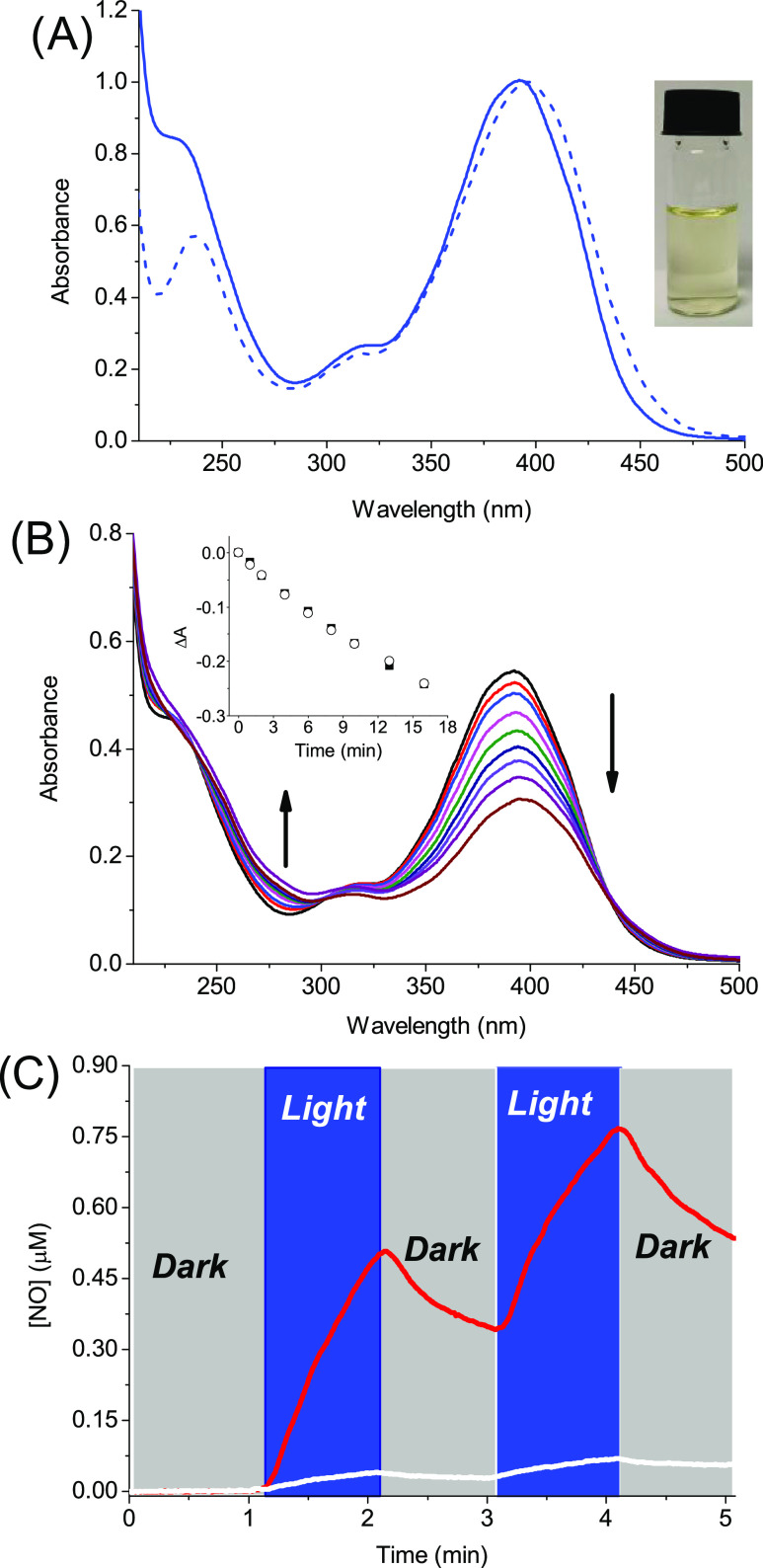
(A) Normalized absorption spectra of **βγCD-NOPD** (solid line) and the model compound **NOPD-1** (dotted line) in water. The inset shows the actual image of the water solution of **βγCD-NOPD**. (B) Absorption spectral changes observed upon light exposure of an air-equilibrated water solution of **βγCD-NOPD** (2 mg mL^–1^) at λ_exc_ = 405 nm for time intervals from 0 to 16 min. The arrows indicate the course of the spectral profile with the illumination time. The inset shows the absorbance changes with the irradiation time at λ = 393 nm observed in air-equilibrated (○) and N_2_-saturated (■) solutions. (C) NO release profile observed for an aqueous solution of **βγCD-NOPD** (2 mg mL^–1^) (red line) and the model compound **NOPD-1** (white line) upon alternate cycles of irradiation (λ_exc_ = 405 nm) and dark. *T* = 25 °C.

**βγCD-NOPD** (*M*_W_*ca.* 70 kD) contains *ca.* 1.8% (w/w) of NOPD and is highly soluble in aqueous media. Figure 1A shows the UV–vis absorption spectra of the polymer and, for the sake of comparison, that of the water-soluble model **NOPD-1** (see Scheme 1). The absorption features of **βγCD-NOPD** are dominated by the large band of the NOPD unit in the blue region with a maximum at 393 nm and extending up to 500 nm. Note that, although the spectral shape profile is basically unchanged, the band of the NOPD moiety in the case of the polymer is shifted to the blue of *ca.* 4 nm if compared with the model compound. Due to the large charge transfer character of this band, the observed shift agrees with a less polar environment experienced by the NOPD moiety, which results in not complete exposure to the water pool but a partial embedding within more hydrophobic compartments of the polymeric network.

Irradiation of **βγCD-NOPD** leads to the bleaching of the main absorption band with a photolysis rate independent of the presence of oxygen (Figure 1B and related inset). This photobehavior is in excellent agreement with that shown by the individual NOPD,^40^ accounting for the loss of NO upon light irradiation and confirming that the integration of the NOPD unit within the polymeric scaffold does not change the nature of the primary photochemical process.

Photostimulated NO release was confirmed by direct amperometric detection. Figure 1C clearly shows that NO production is strictly dependent on the irradiation conditions as demonstrated by the alternate light/dark cycles. Interestingly, we obtained a value for the NO photorelease quantum yield Φ_NO_ = 0.007 ± 0.001, which is more than 1 order of magnitude higher than observed for the model **NOPD-1** under the same irradiation conditions (see traces in Figure 1C). This value is in good agreement with what has been recently observed for NOPDs based on the same chromophore covalently linked to branched polymers based only on βCD units^19,37^ or entrapped in micellar hosts.^42,43^ This enhancement of photoreactivity is the result of the active role of the CD units of the polymer as a reactant, providing H-atoms to the phenoxy radical involved in the NO photorelease mechanism (see Scheme 2).

**Figure 2:**
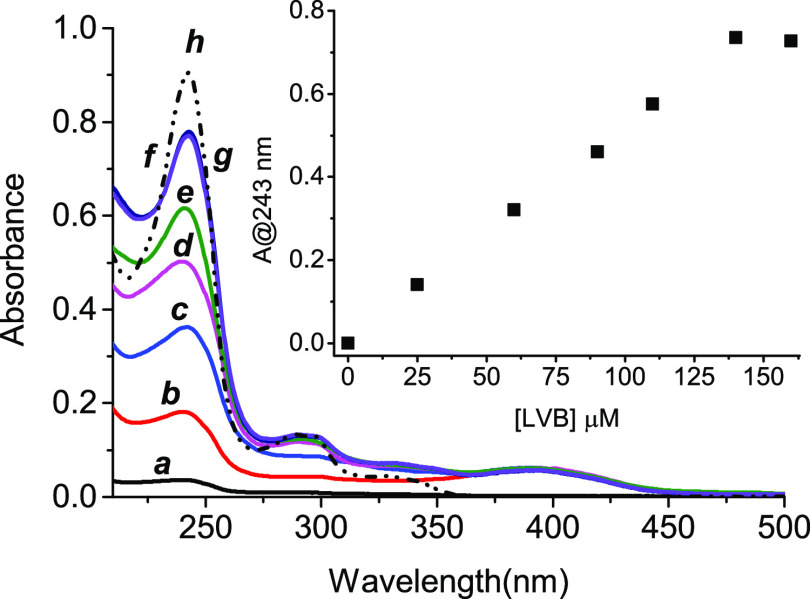
Absorption spectra of **LVB** at 4.1 μM in water (***a***) and in the presence of **βγCD-NOPD** (2 mg mL^–1^) at 25 μM (***b***), 60 μM (***c***), 90 μM (***d***), 110 μM (***e***), 140 μM (***f***), and 160 μM (***g***). The spectrum of a 125 μM **LVB** solution in methanol is shown for the sake of comparison (***h***). The inset shows the absorbance values of **LVB** at λ = 243 nm and at different concentrations in the presence of **βγCD-NOPD** subtracted from the contribution of **βγCD-NOPD**. *T* = 25 °C; cell path = 0.1 cm.

**βγCD-NOPD** emerged as a good host to encapsulate the poorly water-soluble **LVB**, whose solubility in this solvent is ∼4 μM (∼1.7 μg mL^–1^). Figure 2 shows that the drug solubility significantly increases in the presence of the polymer, reaching a value of *ca.* 125 μM (*ca.* 65 μg mL^–1^) (inset Figure 2). Such a value is *ca.* 30-fold higher than that observed in the absence of the polymer and more than 2-fold higher than that observed for the same drug in the presence of the same amount of a branched polymer containing only βCD units.^19^ We obtained values of *ca.* 97% for the encapsulation efficiency and *ca.* 3.3% for drug loading (see the Supporting Information).

Note that the absorption profile of the encapsulated **LVB** is basically identical to that observed in the methanol solution (see spectrum ***h*** in Figure 2), accounting for the lack of any intrahost aggregation of the chemodrug. Moreover, the absorption spectra of Figure 2 in the whole concentration range explored did not change for several days, indicating good stability of the complex under ambient conditions.

**Figure 3:**
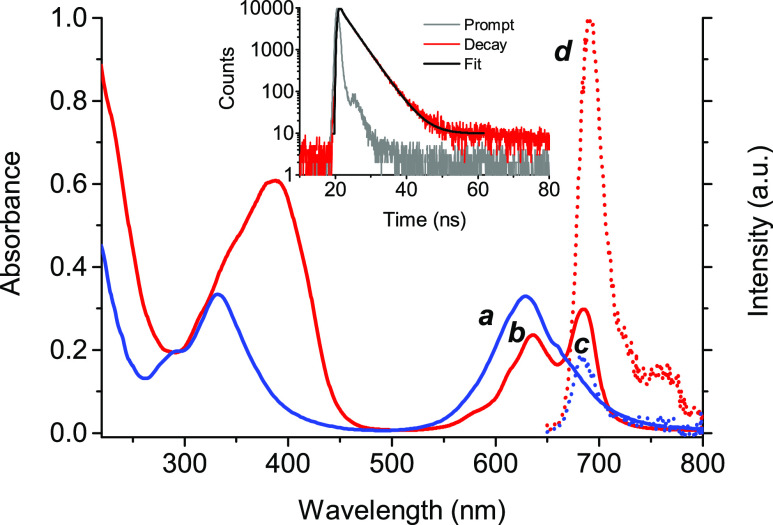
Absorption spectra (solid lines) of **ZnPc** (10 μM) in water (***a***) and in the presence of **βγCD-NOPD** (2 mg mL^–1^) (***b***) and related fluorescence emission spectra (dotted lines) (λ_exc_ = 640 nm) of samples ***a*** (***c***) and ***b*** (***d***). The inset shows the fluorescence decay and the related fitting of **ZnPc** in the presence of **βγCD-NOPD** recorded at λ_exc_ = 635 nm and λ_em_ = 690 nm. *T* = 25 °C.

**βγCD-NOPD** was also a very suitable host to interact with the highly hydrophilic **ZnPc** (see Scheme 1). This compound is a well-known PS for PDT, being able to generate the cytotoxic ^1^O_2_ for therapy and to emit red fluorescence useful for imaging under red-light excitation.^44^**ZnPc** is very soluble in aqueous solution, where it shows absorption bands at 335 and 635 nm, respectively (spectrum ***a*** in Figure 3). However, the formation of water-soluble aggregates^45^ precludes its response to light, resulting in the ineffective population of the triplet state, consequent lack of ^1^O_2_ photogeneration, and very low fluorescence emission in this solvent.^46,47^ Addition of **βγCD-NOPD** to a water solution of **ZnPc** significantly breaks its self-aggregation, encouraging the entangling of the PS within the polymeric network as a monomer in a satisfactory amount (≥20%). This is confirmed by the decrease of the absorption band of the aggregate form at 635 nm and the concomitant formation of a new absorption band with λ_max_ = 680 nm, typical for the monomeric species^45^ (spectrum ***b*** in Figure 3). In addition, a significant increase in the typical red emission of **ZnPc** when compared to that in the absence of polymer was observed (spectra ***c*** and ***d*** in Figure 3). The fluorescence decay is characterized by a dominant component (≥84%) with the lifetime τ = 3.5 ns (inset Figure 3). Note that the emission spectra observed in the presence of the polymer are red-shifted *ca.* 7 nm when compared with the free **ZnPc**. This is the result of environmental effects on the photophysical features of the PS, in accordance with its red-shifted emission observed in nonaqueous media compared to water solution.^48^ As previously reported, the entrapment of momomeric **ZnPc** can be encouraged by the three-dimensional (3D) structure of the CD polymer in which the high local concentration of CD nanocavities in the cross-linked network may act cooperatively in the disruption of the aggregate forms.^48^ In principle, **ZnPc** is able to interact with the polymer considering that the benzene sulfonate moieties attached to its macrocyclic structure are available to interact with the β and γCD units due to their favorable geometrical matching.^49,50^

In addition to restoring the fluorescence emission of **ZnPc**, the branched polymeric host leads to the population of the lowest triplet state of the PS, which is a key transient intermediate involved in the photogeneration of the cytotoxic ^1^O_2_ through collisional energy transfer with molecular oxygen.^7,9^ Nanosecond laser flash photolysis is a powerful tool to obtain direct evidence on spectroscopic and kinetic features of the triplet states of the porphyrinoid systems. They show in fact intense absorptions in the visible region and have lifetimes falling in the microsecond time regime.^51^ In contrast to the negligible signal observed in neat water, laser excitation of **ZnPc** in the presence of **βγCD-NOPD** shows the appearance of the characteristic triple–triplet absorption of **ZnPc** characterized by a maximum at 500 nm and a bleaching in the correspondence of the ground state absorption of the monomeric form at 680 nm (Figure S9). The triplet decay is biexponential with lifetimes τ_1_ ∼ 20 μs and τ_1_ ∼ 300 μs (Figure S10), which probably reflect different triplet population confined in different regions of the host.

### 2.2 βγCD-NOPD and Its Ternary Assembly with LVB and ZnPc

**Figure 4:**
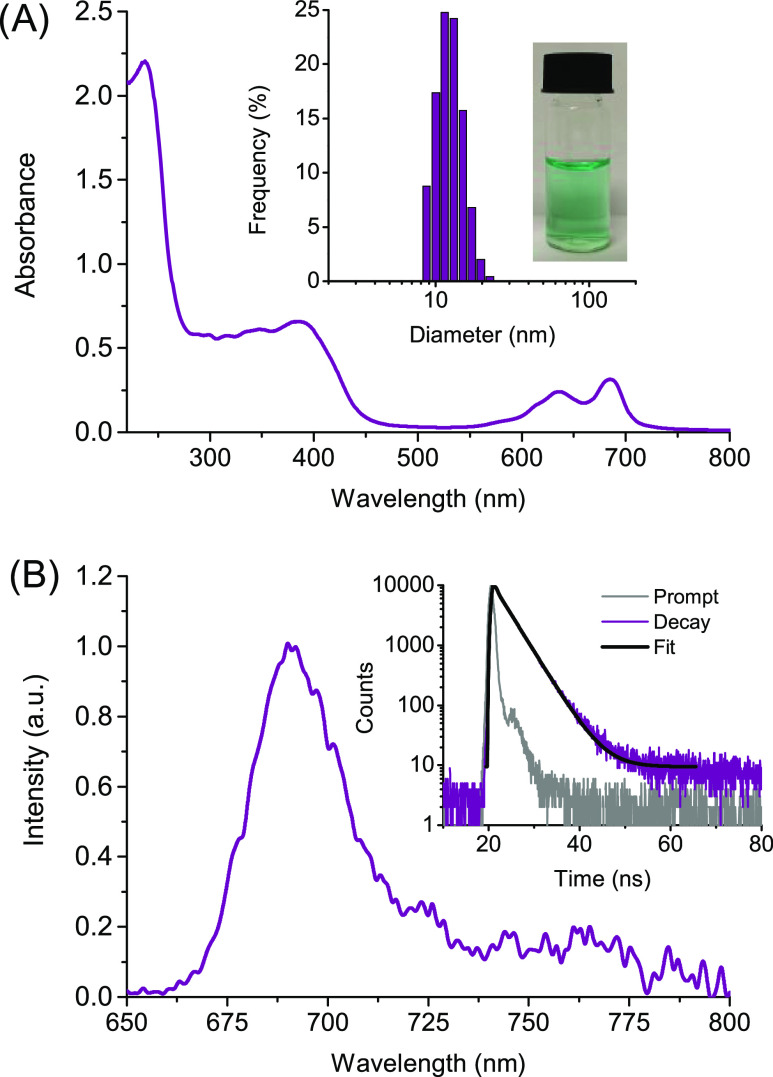
(A) Absorption spectrum of the aqueous solution of **βγCD-NOPD** (2 mg mL^–1^) loaded with **LVB** (25 μM) and **ZnPc** (10 μM). The inset shows the hydrodynamic diameter and the actual image of the ternary supramolecular assembly. (B) Fluorescence emission spectrum of the sample reported in (A) (λ_exc_ = 640 nm). The inset shows the fluorescence decay and the related fitting recorded at λ_exc_ = 635 nm and λ_em_ = 690 nm. *T* = 25 °C.

Both **LVB** and **ZnPc** were co-encapsulated in **βγCD-NOPD** in two steps (see the Supporting Information). Figure 4A reports the spectrum of the supramolecular ensemble where the typical absorption of **βγCD-NOPD** at *ca.* 390 nm is accompanied by the characteristic absorption bands of **LVB** at *ca.* 240 nm and the partially monomerized **ZnPc** at *ca.* 680 nm. Note that the absorbance values, absorption maxima position, and spectral profile of all chromogenic components are basically not changed with respect to those observed when the guests are individually encapsulated in the polymeric host (compare Figure 4A with Figures 2 and 3). This finding indicates that the co-encapsulation does not lead to a significant displacement or aggregation of the individual chromogenic components and is probably the result of their different affinity for the multiple binding sites of the polymeric host. Dynamic light scattering measurements (inset Figure 4) gave an average hydrodynamic diameter for the supramolecular assembly of *ca.* 15 nm. This value is slightly larger than that observed for the free host (∼11 nm), according to what has been already observed for similar branched polymers after the supramolecular encapsulation of multiple guests.^34,35^ In addition, the fluorescence properties of **ZnPc** when co-encapsulated with **LVB** were identical to those already found when it is individually entangled in the polymer, as confirmed by the same emission efficiency and fluorescence lifetime (Figure 4B and related inset).

The photodynamic properties of a multicargo nanoassembly were then investigated in terms of the capability to generate NO and ^1^O_2_ under visible-light excitation.

**Figure 5:**
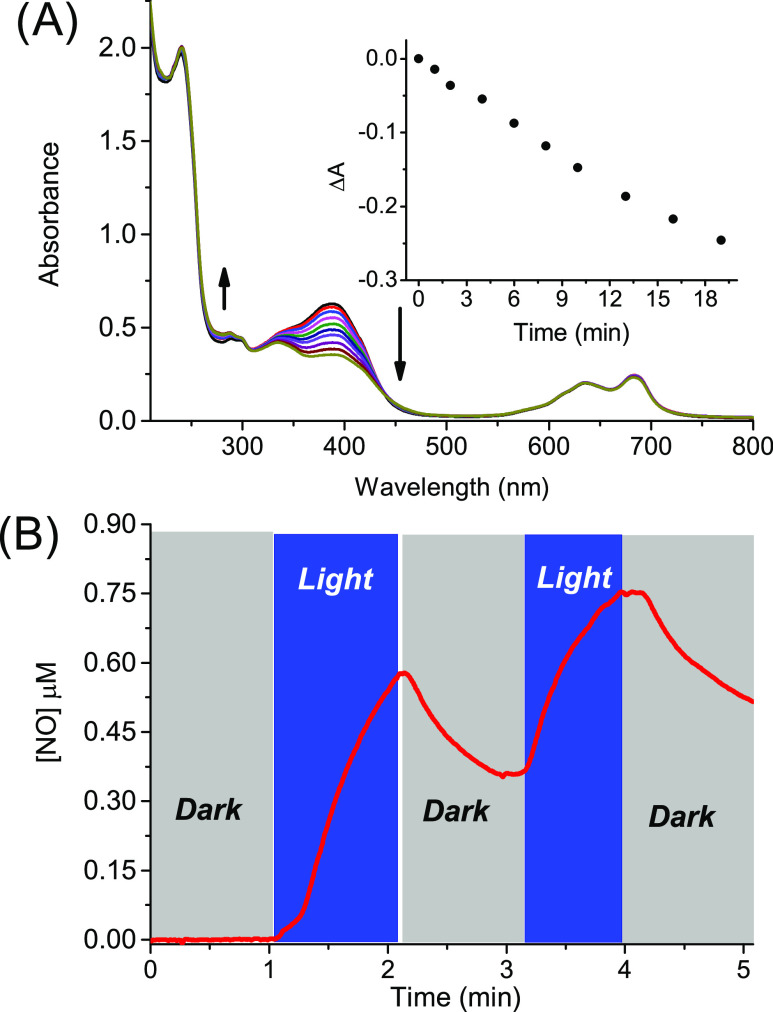
(A) Absorption spectral changes observed upon light exposure of an air-equilibrated water solution of **βγCD-NOPD** (2 mg mL^–1^) loaded with **LVB** (25 μM) and **ZnPc** (10 μM) at λ_exc_ = 405 nm for time intervals from 0 to 19 min. The arrows indicate the course of the spectral profile with the illumination time. The inset shows the absorbance changes with the irradiation time at λ = 393 nm. (B) NO release profile observed for a sample as in (A) upon alternate cycles of irradiation (λ_exc_ = 405 nm) and dark. *T* = 25 °C.

Irradiation of the polymeric nanoassembly with blue light induces bleaching of the main absorption band of the NOPD moiety at *ca.* 390 nm (Figure 5A), with a photolysis profile and photodecomposition rate (inset Figure 5A) very similar to those observed for the free polymeric host (see Figure 1B and related inset for comparison) and accounting well for the NO release. Again, this was unambiguously confirmed by the direct NO detection (Figure 5B), which showed the photogeneration of NO occurring with the same efficiency as observed for the empty host **βγCD-NOPD** (see Figure 1C for the sake of comparison). These results suggest that the co-presence of **LVB** and **ZnPc** within the polymeric network does not induce either quenching phenomena or unexpected photochemical reactions competitive with the NO release. This is further confirmed by the lack of spectral changes observed in the correspondence of the absorption bands of **LVB** and **ZnPc** in the UV and red region, respectively (see Figure 5A), in accordance with the preservation of the structural integrity of these components after light irradiation.

**Figure 6:**
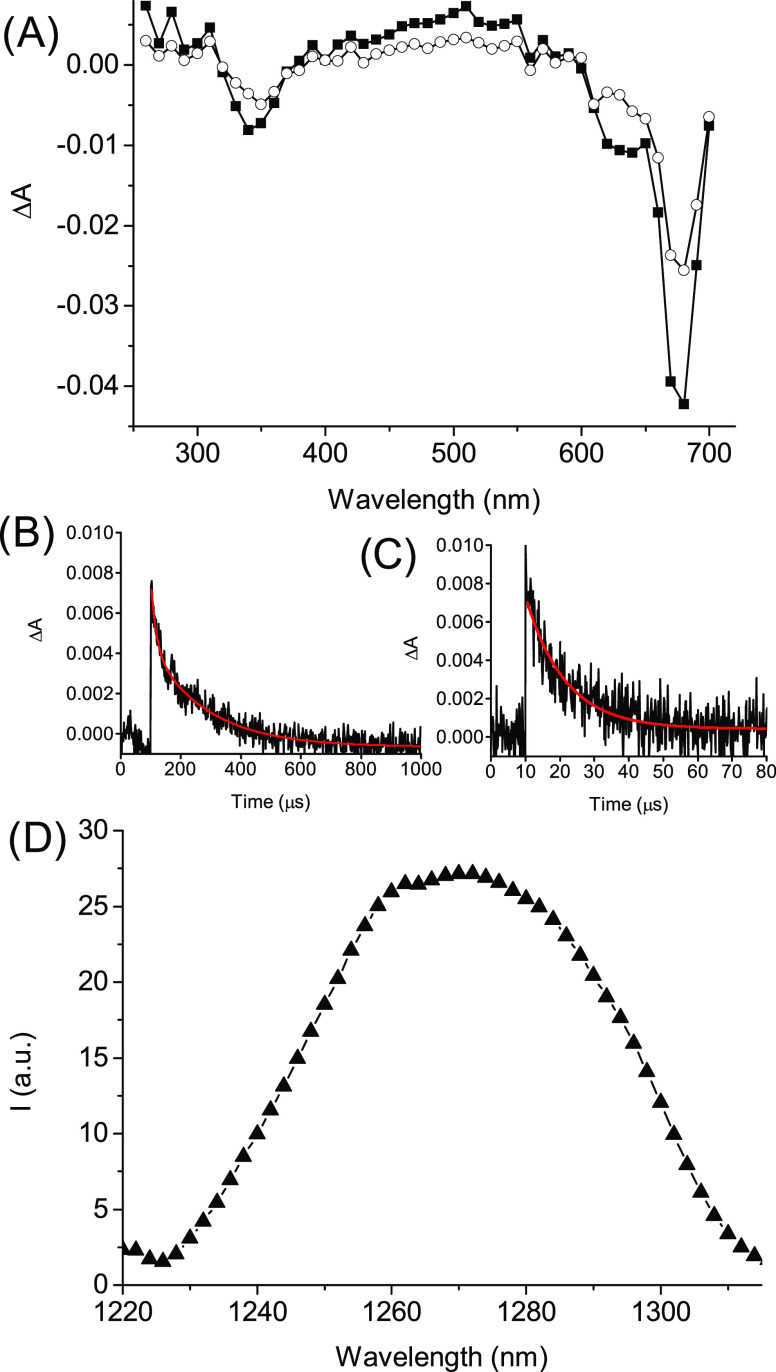
(A) Transient absorption spectra observed 1 μs (■) and 80 μs (○) after 355 nm laser excitation (*E*_355_ ∼ 10 mJ/pulse) of the N_2_-saturated water solution of **βγCD-NOPD** (2 mg mL^–1^) loaded with **LVB** (25 μM) and **ZnPc** (10 μM). (B) Decay trace and related biexponential fitting of the same sample as in (A) monitored at 500 nm under N_2_-saturated conditions. (C) Decay trace and related monoexponential fitting of the same sample as in (A) monitored at 500 nm under air-equilibrated conditions. (D) ^1^O_2_ luminescence detected upon 671 nm light excitation of an air-equilibrated D_2_O solution of **βγCD-NOPD** (2 mg mL^–1^) loaded with **LVB** (25 μM) and **ZnPc** (10 μM). *T* = 25 °C.

Figure 6A shows the transient absorption spectra recorded after the initial laser pulse. The spectrum observed at 1 μs shows a maximum at 500 nm and bleaching at 680 nm, typical for the lowest triplet state of the monomeric form of **ZnPc**. The triplet absorbance is comparable to that observed in the absence of **LVB** (see Figure S9 for the sake of comparison). By taking into account the fact that the two solutions have the same absorbance at the excitation wavelength and that the molar extinction coefficient of the triplet state is not expected to significantly change, basically keeping their band profiles unchanged, the triplet absorbance can be directly related to the efficiency of the triplet population which, therefore is not affected by the co-presence of **LVB**. The chemodrug has no influence on the dynamic of the **ZnPc** triplet. The time evolution of this species with the elapsing time reveals in fact no additional transients formed concurrently to its decay (spectrum at 80 μs, Figure 6A), ruling out any possible bimolecular reaction. Analogously to what has been already observed in the absence of **LVB** (Figure S10), the **ZnPc** triplet decay was biexponential with lifetimes of τ_1_ ∼ 18 μs and τ_2_ ∼ 200 μs (Figure 6B). The triplet state is effectively quenched by oxygen as demonstrated by the significant shortening of the triplet decay, which, under air-equilibrated conditions, was monoexponential with a lifetime of τ ∼ 10 μs (Figure 6C). This finding provides direct evidence that despite entanglement within the polymeric network, the triplet state of the PS is still accessible to molecular oxygen for the collisional energy transfer, which is crucial for ^1^O_2_ generation.

^1^O_2_ formation upon red-light excitation of the nanoassembly was unambiguously demonstrated by its diagnostic phosphorescence spectrum in the near-IR region with a maximum at *ca*. 1270 nm^9^ (Figure 6D). A value for the ^1^O_2_ quantum yield Φ_Δ_ = 0.25 ± 0.05 was obtained using methylene blue as a standard (see the Supporting Information). Note that the red-light irradiation under aerobic conditions does not lead to any significant changes in the whole absorption spectrum. This confirms not only the absence of any undesired bimolecular reaction of the triplet state of **ZnPC** with both the NOPD unit of the polymer and the co-encapsulated **LVB** but also rules out any potential oxidation of all chromogenic components by the photogenerated ^1^O_2_.

## 3 Conclusions

We have synthesized a branched polymeric material covalently integrating both β and γCD units together with an NOPD within its macromolecular network. The photoresponsive **βγCD-NOPD** polymer is highly soluble in water, offers compartments with different sizes and hydrophobicities and represents a versatile polymeric host for the straightforward modular integration of multiple functional components. In fact, the simultaneous co-encapsulation of a hydrophobic chemodrug and a hydrophilic PS allows to achieve a system with multiple photo-chemotherapeutic cargos into a single supramolecular construct with functions that would be otherwise impossible to replicate with the separate components.

In particular, the polymeric host (i) amplifies the photoreleasing efficiency of the cytotoxic NO due to its active role as a reactant in the NO photorelease process; (ii) increases the solubility of **LVB** by more than 30-fold if compared with the free drug and more than 2-fold if compared with a similar polymer containing only βCD units; and (iii) disrupts the aggregation of the non-photoresponsive **ZnPc**, making it photochemically active and able to produce the cytotoxic ^1^O_2_ and to emit red fluorescence.

A remarkable point of this nanoconstruct is the absence of mutual interactions between the NOPD unit, the PS, and the chemodrug both in the ground and excited states, despite all components confined in the same host. This result is not trivial and allows the preservation of nature and efficiency of the individual photochemical properties of the phototherapeutic components while preserving the structural integrity of the chemodrug. In view of these results, we believe this polymeric multicargo nanoplatform, the first to our knowledge with the described peculiarities, may represent an intriguing system with potential trimodal photo-chemotherapeutic action to be tested in biological systems.

## Supporting Information Available

Synthesis and characterization of **βγCD-NOPD** and transient spectrum and time decay of the triplet state of **ZnPc** encapsulated in the polymer (PDF)
