# Supplementary material for: Mixed β-γ-Cyclodextrin Branched Polymer with Multiple Photo-Chemotherapeutic Cargos
Source: ACS Appl Polym Mater. 2023 Aug 31;5(10):7918–26. doi: 10.1021/acsapm.3c01157 (PMC10580695; doi:10.1021/acsapm.3c01157)
Supplement: Supplementary file 1 — ap3c01157_si_001.pdf [file ap3c01157_si_001.pdf]

Supporting Information

A mixed β-γ-cyclodextrin branched polymer with multiple photo-chemotherapeutic cargos

Francesca Laneri, †,# Mimimorena Seggio,†,§,# Cristina Parisi,† Szabolcs Béni,Π Aurore Fraix,† Milo Malanga,‡,¥ and Salvatore Sortino†,*

†PhotoChemLab, Department of Drug and Health Sciences, University of Catania, I-95125 Catania, Italy.

ΠDepartment of Pharmacognosy, Semmelweis University, I-1085 Budapest, Hungary.

‡CycloLab, Cyclodextrin R&D Ltd, I-1097 Budapest, Hungary.

§Present address: Department of Biotechnology, University of Verona, I-37134, Verona, Italy.

¥Present address: CarboHyde, I-1045 Budapest, Hungary.

#Contributed equally

*Corresponding author: ssortino@unict.it

Materials.

βγCD-NOPD synthesis is reported in SI. **ZnPc** was purchased from Porphyrin Pdts and used without further purification. **LVB** mesylate was purchased from MedChemExpress (HY-10981, Madrid, Spain). The model compound **NOPD-1** was synthesized according to our previously reported procedure.[S1] All other reagents (Sigma-Aldrich) were of high commercial grade and were used as received. All solvent used (from Carlo Erba) were spectrophotometric grade. The polymer was solubilized in MilliQ water which was used for all the chemical and photochemical experiments.

Sample preparation.

Solutions of **βγCD-NOPD** were prepared by stirring overnight 2 mg mL^{-1} of polymer in water. **ZnPc** was dissolved in water, then added to 2 mg mL^{-1} of **βγCD-NOPD** and stirred at room temperature for 24 h. The concentration of **ZnPc** in water was obtained by UV-Vis spectroscopy, using a molar extinction coefficient of 31.000 M^{-1} cm^{-1}.[S2] **LVB** was solubilized in methanol and the obtained solution was then gently evaporated to obtain a thin film. Afterwards, the film was hydrated with aqueous solutions of **βγCD-NOPD** without or with **ZnPc**. The mixtures were stirred for 5 hours at room temperature in the dark and then filtered. The concentration of **LVB** was obtained spectrophotometrically, using molar extinction coefficients of 72.350 M^{-1}

cm^{-1} at λ = 241 nm and 62.100 M^{-1} cm^{-1} at λ = 245 nm for the free drug in MeOH and when complexed with **βγCD-NOPD**, respectively. Encapsulation efficiency (EE %) and loading capacity (LC %) were calculated as below:

$$EE\ \% = (W_{IN}/W_i) \times 100$$

$$LC\% = W_{IN} / (W_{IN} + W_P) \times 100$$

where W_{IN} is the amount of **LVB** in the **βγCD-NOPD**, Wi is the total amount of **LVB** added initially during preparation, and W_P is the amount of **βγCD-NOPD**.

Instrumentation.

^{1}H-NMR and ^{13}C spectra were recorded on a Varian UNITY Inova at 500 MHz. Dynamic and static light scattering measurements were performed with a zetasizer Nano ZS Malvern. UV-Vis spectra absorption and fluorescence emission spectra were recorded with a JascoV-560 spectrophotometer and a Spex Fluorolog-2 (mod. F-111) spectrofluorimeter, respectively, in air-equilibrated solutions, using quartz cells with a path length of 1 cm. Fluorescence lifetimes were recorded with the above fluorimeter equipped with a TCSPC Triple Illuminator. The samples were excited with a pulsed diode excitation source (Nanoled) at 635 nm, the decays were monitored at 690 nm, and ethanol solution itself was used to register the prompt at 635 nm. The system allowed a time-resolution > 200 ps. The multiexponential fit of the fluorescence decay was obtained by the following equation:

$$I(t) = \Sigma\alpha_i \exp(^{-t/\tau_i})$$

Absorption spectral changes were monitored by irradiating the sample in a thermostated quartz cell (1 cm path length, 3 mL capacity unless specified) under gentle stirring, using a continuum laser with λ_{exc} = 405 nm, *ca.* 100 mW, having a beam diameter of *ca.* 1.5 mm.

NO detection. Direct monitoring of NO release in solution was performed by amperometric detection (World Precision Instruments), with an ISO-NO meter, equipped with a data acquisition system, and based on direct amperometric detection of NO with short response time (< 5 s) and sensitivity range 1 nM – 20 µM. The analogue signal was digitalized with a four-channel recording system and transferred to a PC. The sensor was accurately calibrated by mixing standard solutions of $NaNO_2$ with 0.1 M H_2SO_4 and 0.1 M KI according to the reaction:

$$4H^+ + 2I^- + 2NO_2^- \rightarrow 2H_2O + 2NO + I_2$$

Irradiation was performed in a thermostated quartz cell (1 cm path length, 3 mL capacity) using the continuum laser with λ_{exc} = 405 nm. NO measurements were carried out under stirring with the electrode positioned outside the light path in order to avoid NO signal artefacts due to photoelectric interference on the ISO-NO electrode.

$^1O_2$ detection. Direct photogeneration of $^1O_2$ was monitored by its typical near infrared (NIR) luminescence resulting from the forbidden transition $^3\Sigma_g^- \leftarrow {}^1\Delta_g$; the measurements of luminescence were performed with the same spectrofluorimeter equipped with a NIR-sensitive liquid nitrogen cooled photomultiplier exciting the air-equilibrated samples with a 671 nm continuum laser (200 mW).

Laser flash photolysis. All of the samples were excited with the third harmonic of Nd–YAG Continuum Surelite II–10 laser (355 nm, 6 ns FWHM), using quartz cells with a path length of 1.0 cm. The excited solutions were analyzed with a Luzchem Research mLFP–111 apparatus with an orthogonal pump/probe configuration. The probe source was a ceramic xenon lamp coupled to quartz fiber-optic cables. The laser pulse and the mLFP–111 system were synchronized by a Tektronix TDS 3032 digitizer, operating in pre-trigger mode. The signals from a compact Hamamatsu photomultiplier were initially captured by the digitizer and then transferred to a personal computer, controlled by Luzchem Research software operating in the National Instruments LabView 5.1 environment. The solutions were deoxygenated by bubbling with a vigorous and constant flux of pure argon (previously saturated with solvent). In all of these experiments, the solutions were renewed after each laser shot (in a flow cell of 1 cm optical path), to prevent probable auto-oxidation processes. The sample temperature was 295 ± 2 K. The energy of the laser pulse was measured at each shot with a SPHD25 Scientech pyroelectric meter.

NO photorelease and $^1O_2$ photogeneration quantum yields.

NO photorelease quantum yield (Φ_{NO}) was determined at λ_{exc} = 405 nm within the 20% transformation of **βγCD-NOPD** by using the following equation

$$\Phi_{NO} = [C]xV\ /t\ x(1-10^{-A})xI$$

where [C] is the concentration of phototransformed **βγCD-NOPD**, V is the volume of the sample, t is the irradiation time, A is the absorbance of the sample at the excitation wavelength and I the intensity of the excitation light source. The concentration of the phototransformed **βγCD-NOPD** was determined both spectrophotometrically, by taking into account the absorption changes at 400 nm and a $\Delta\varepsilon_{393}$ = 10.000 M^{-1} cm^{-1}; I was calculated by potassium ferrioxalate actinometry.

$^1O_2$ photogeneration quantum yield (Φ_Δ) was determined in D_2O solution using optically-matched samples at the excitation wavelength of **ZnPc** encapsulated within **βγCD-NOPD** and methylene blue in the same solvent as standard (Φ_Δ = 0.50).[S3] The values of Φ_Δ were determined from the following equation:

$$\Phi_{\Delta} = \Phi_{\Delta}(s) \times (I/I(s))$$

where $\Phi_{\Delta}(s)$ is the $^1O_2$ quantum yield of the standard, I and I(s) are the areas of the fluorescence spectra of compounds and standard, respectively.

Syntheses and characterization

Synthesis of azidated-NOPD (3). In order to selectively modify the cyclodextrin scaffold on the primary rim, the NOPD containing synthon was prepared according to the following three steps strategy (Scheme S1).

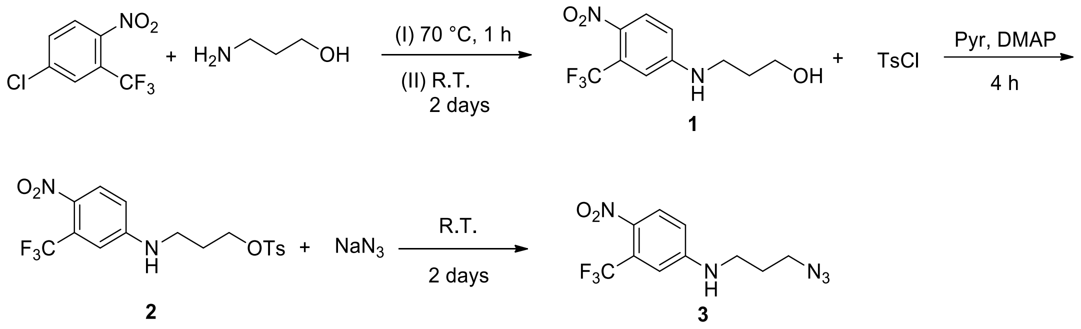

Scheme S1. Synthetic strategy towards azidated-NO-donor (compound **3**).

Briefly, 5-cloro-2-nitrobenzotrifluoride (4.5 g, 20.0 mmol) was heated at 70 °C for 1 h with an excess of 3-amino-1-propanolo (17.7 g, 235.6 mmol, 18 mL). The reaction was cooled down and was stirred at room temperature for 2 days. The solvent was removed under reduced pressure and the residue was precipitated with water. The solid was filtered-off using a glass filter (porosity 3) and it was dried in a vacuum drying box until constant weight (5 g, 90% yield). The obtained intermediate, compound **1** (3 g, 11.5 mmol), was solubilized in pyridine (50 mL), cooled down to 0 °C and 4-toluenesulfonyl chloride (2.16 g, 11.3 mmol) and DMAP (0.55 g) were added in sequence. The reaction mixture was stirred overnight. The solvent was removed under reduced pressure and the residue was solubilized in CH_2Cl_2 (30 mL) and extracted with water (3 x 30 mL). The organic phase was concentrated and the viscous residue was purified by chromatography (Hexan:EtOAc = 7:3) obtaining compound (**2**) as yellow powder (4.5 g, 97% (**2**). Compound **2** (4 g, 9.6 mmol) was dissolved in DMF/DMSO 1:1 (60 mL), sodium azide was added in one portion (1.55 g, 24 mmol) and left under stirring for 2 days at 60 °C. The reaction mixture was diluted with water (120 mL) and extracted with CH_2Cl_2 (3 x 120 mL). The organic phases were combined and evaporated until dryness yielding the target compound as yellow oil (3 g, 83% yield). ^1H NMR (500 MHz, D_2O, 298 K) δ(ppm) 7.85 (d, 1H, H3a), 6.82 (d, 1H, H2a), 6.51 (d, 1H, H1a), 3.31 (t, 2H, H1), 3.14 (t, 2H, H3), 1.76 (q, 2H, H2).

$(NOPD)_2$-NH-BCD (5). In order to implement the azidated-NO-donor compound on the cyclodextrin scaffold, 6-monoamino-β-CD was first modified with two propargyl moieties according to Scheme S2. 6-monoamino-

β-CD free base (2.3 g, 2.0 mmol) was solubilized in anhydrous DMSO (50 mL). After complete solubilization, DIPEA (2.58 g, 20.0 mmol, 3.5 mL) and propargyl bromide (~80% in toluene, 0.88 g, 5.9 mmol, 0.6 mL) were added in sequence and the mixture was heated at 40 °C for 3 hours. The reaction mixture was concentrated under reduced pressure and the obtained syrup was precipitated with acetone. The solid was filtered-off, extensively washed with acetone and finally dried until constant weight in a vacuum drying box. The crude was additionally purified by chromatography; eluent THF : NH_3 water solution 25% : MeOH = 10:4:1. Fractions were combined and evaporated yielding compound **4** as white powder (1.5 g; 56% yield). MALDI MS: for $C_{48}H_{75}NO_{34}$ calcd m/z 1209.420 found 1210.510 $[M+H]^+$, ^1H NMR (500 MHz, DMSO-d_6) δ(ppm) 5.9-5.74 (m, 7H, C3OH) 4.83-4.39 (m, 7H, H1), 4.46 (m, 2H, H9'), 3.69-3.20 (m, 48H, c), 3.36 (s, 2H, a), 3.27 (s, 2H, b); ^{13}C NMR (125 MHz, DMSO-d_6) assignment based on DEPT-edited HSQC spectrum δ 104.98 (C1), 86.91-73.66 (C2, C3, C4, C5), 102.55-101.08 (C1), 63.27 (C6), 51.78 (Ca), 45.50 (Cc), 42.96 (Cb).

The azide-alkyne cycloaddition reaction between the compounds **3** and **4** was performed as follow. Compound **3** (3 g, 10.4 mmol) and **4** (2 g, 1.65 mmol) were dissolved in DMF/H_2O 2:1 (100 mL) and then Cu(I)Br (0.25 g) was added to the mixture as catalyst. The reaction was stirred at room temperature for 30 minutes. The solvent was partially removed under reduced pressure and the residue was precipitate with acetone. The obtained precipitate was filtered-off, washed with acetone (3x100 mL) until constant weight in a vacuum drying box. The crude was purified by chromatography using as eluent 1,4-dioxane:NH_3 aqueous solution 25%:1-propanol = 10:7:3, yielding **5** as a yellow powder (2.3 g, 1.3 mmol, 78% yield).

The MALDI, the ^1H NMR and HSQC spectra of compound **5** are reported in Figures S1, S2 and S3 respectively.

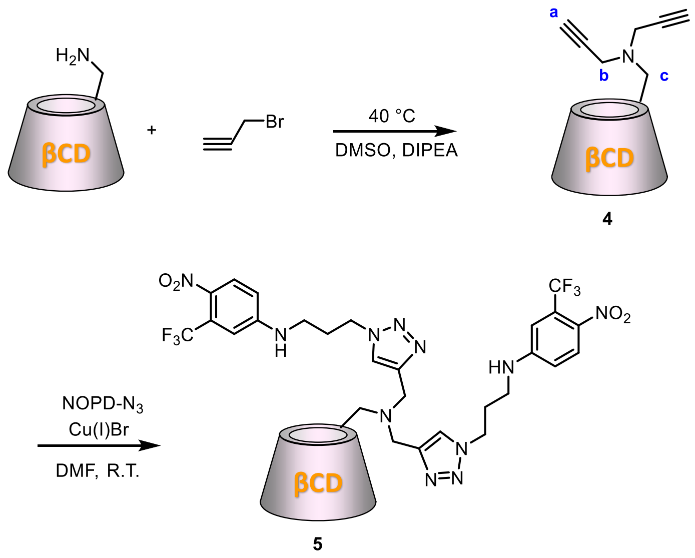

Scheme S2. Synthetic strategy towards NO-donor appended βCD.

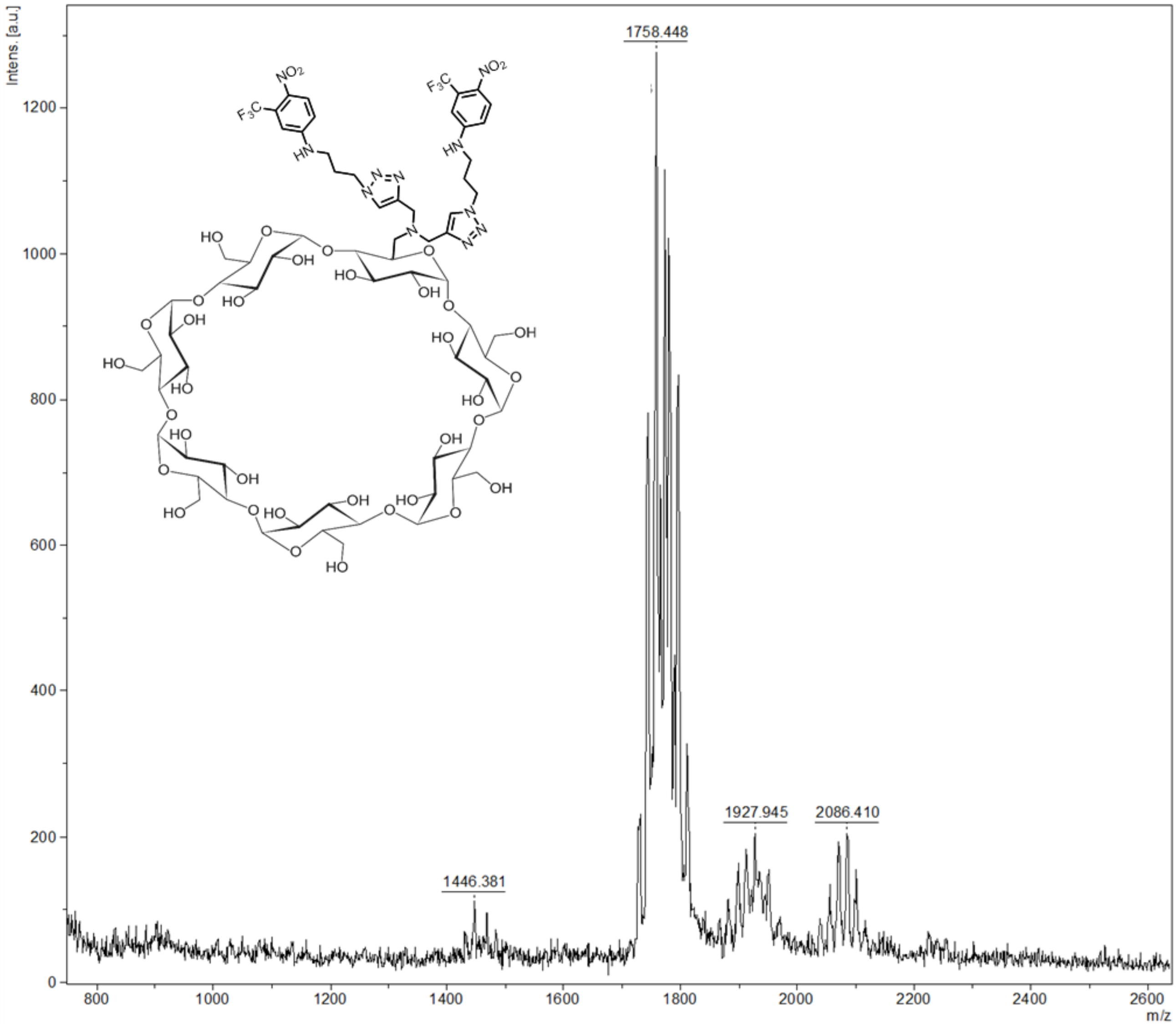

Figure S1. MALDI spectrum of compound **5**.

The found molecular weight differs from the expected one (1790.539 $[M+H]^+$) due to the decomposition of nitro groups into amino groups caused by the laser desorption ionization.

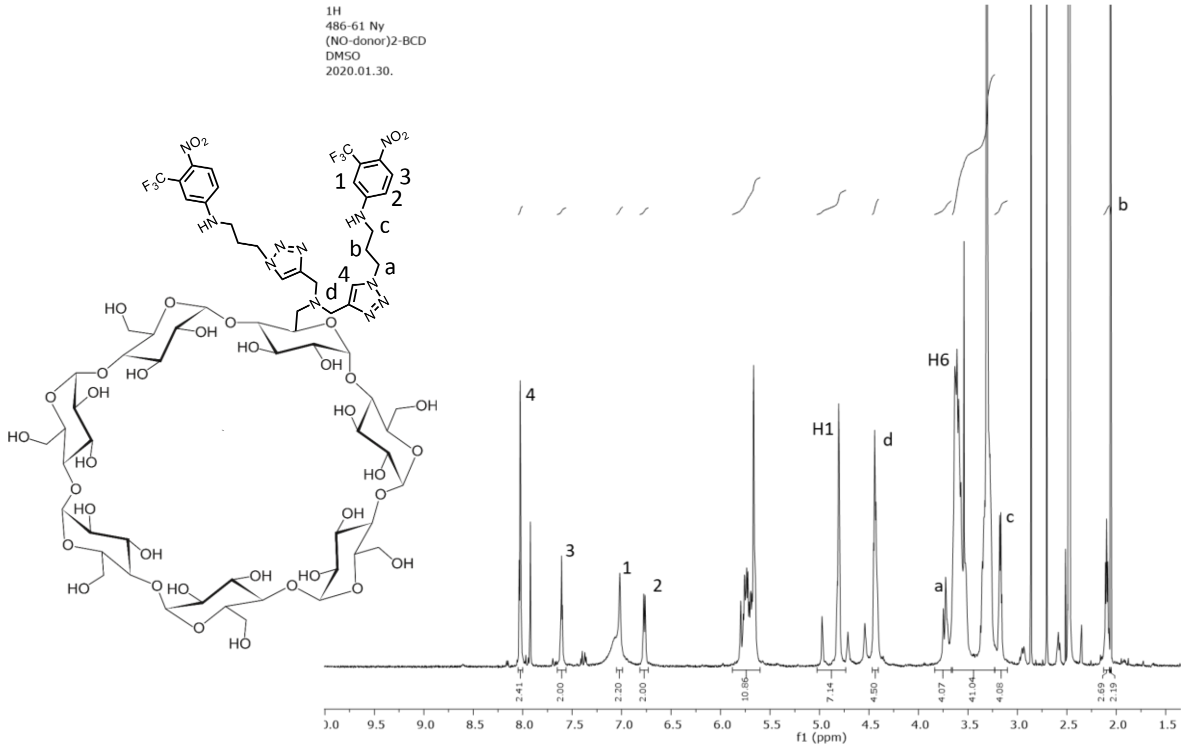

Figure S2. ^{1}H spectrum of compound **5** with partial assignment (DMSO-d_6, 600 MHz, 298 K).

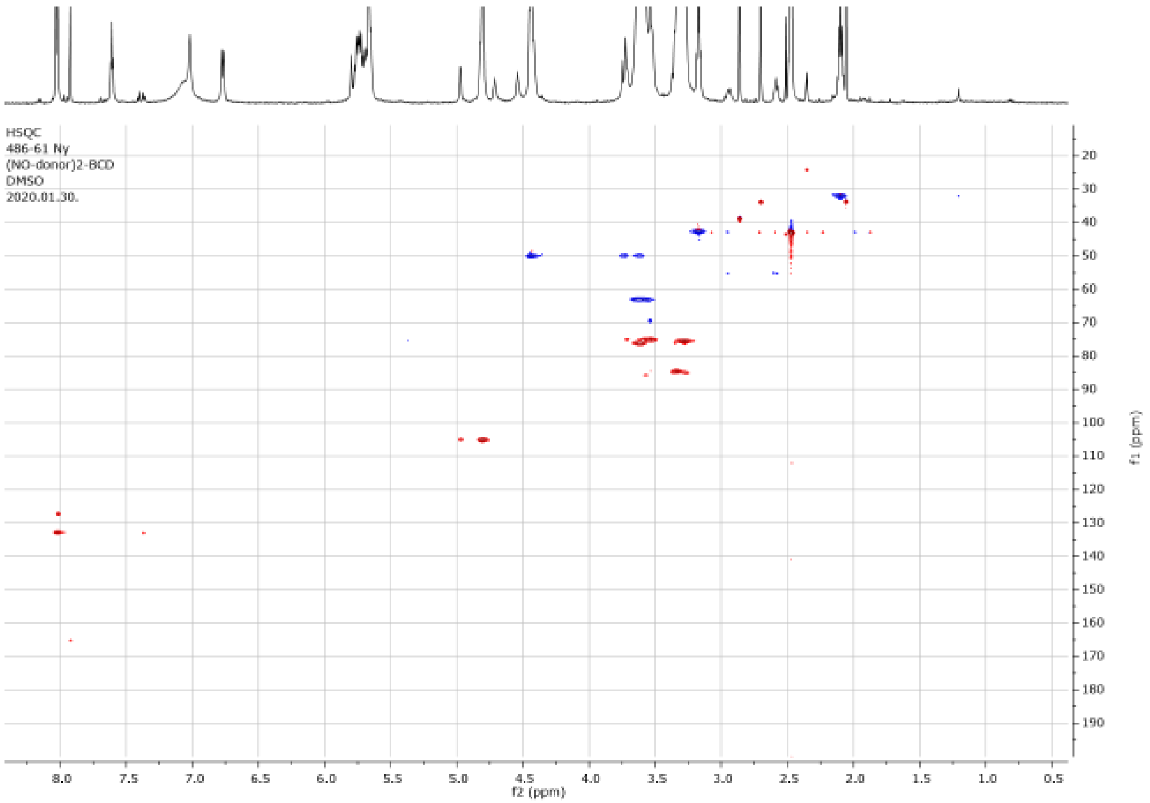

Figure S3. DEPT-edited HSQC spectrum of compound **5** (DMSO-d_6, 600 MHz, 298 K).

Polymerization. Compound **5** (1.5 g) and γ-CD (12 g) was solubilized in 25 mL of an aqueous solution of NaOH 2M. and epichlorohydrin (5 mL) was added dropwise (60 minutes) by keeping the temperature constant at 60 °C (Scheme S3). The reaction mixture was stirred at 60 °C for additional 2 hours, cooled down to 30 °C and left rest overnight at room temperature without stirring. The reaction crude was neutralized with HCl solution 1 N (10 mL) filtered to remove insoluble residue (glass filter porosity 3), dialyzed and freeze dried. The NOPD unit for compound **5** was estimated 1.8% based on analysis of the absorption spectrum. The characterization of the final polymer by NMR was reported in Figures S4, S5, S6, S7 and S8.

The **βγCD-NOPD** molecular weight determination was performed using the Static Light Scattering (SLS) method and a value of 70 kDa ± 5 (PDI = 0.5) was obtained. The measurements were realized according to the protocol reported by Puskàs et al.[S4] The stock solution was filtered (syringe filter at 0.22 μm) prior the use, in order to remove self-assembled aggregates. The polymer sample was studied at concentrations ranging from 1 to 10 mg mL^{-1} at a temperature of 25 °C using toluene as a reference and a differential refractive index value of d_n/d_c=0.14. The measurement was repeated three times and the experimental values were plotted in order to optimize the statistic parameter. The obtained correlation coefficient of the Debye plots was higher than 0.9.

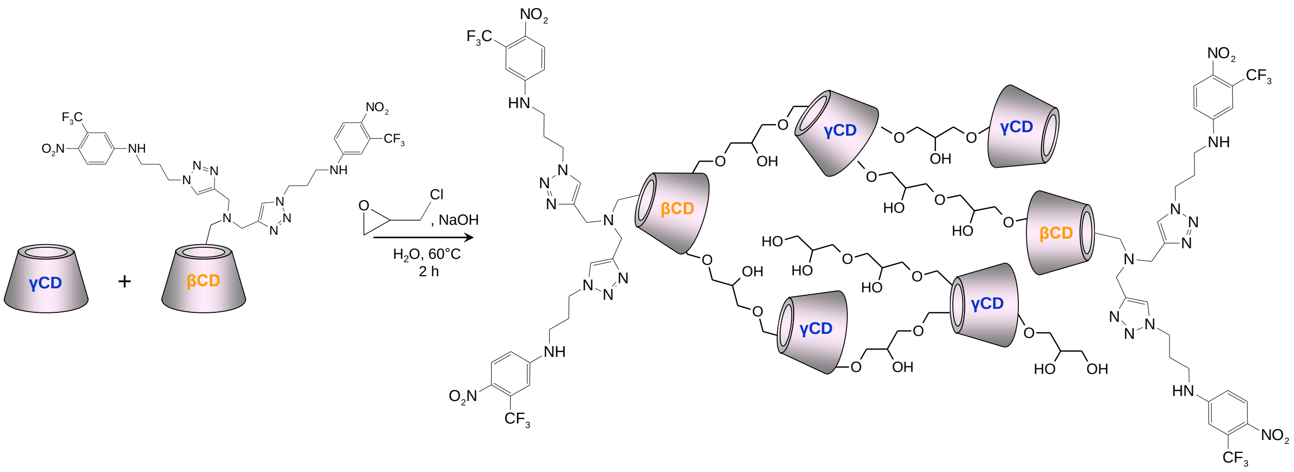

Scheme S3. Synthetic strategy towards polymer **βγCD-NOPD**.

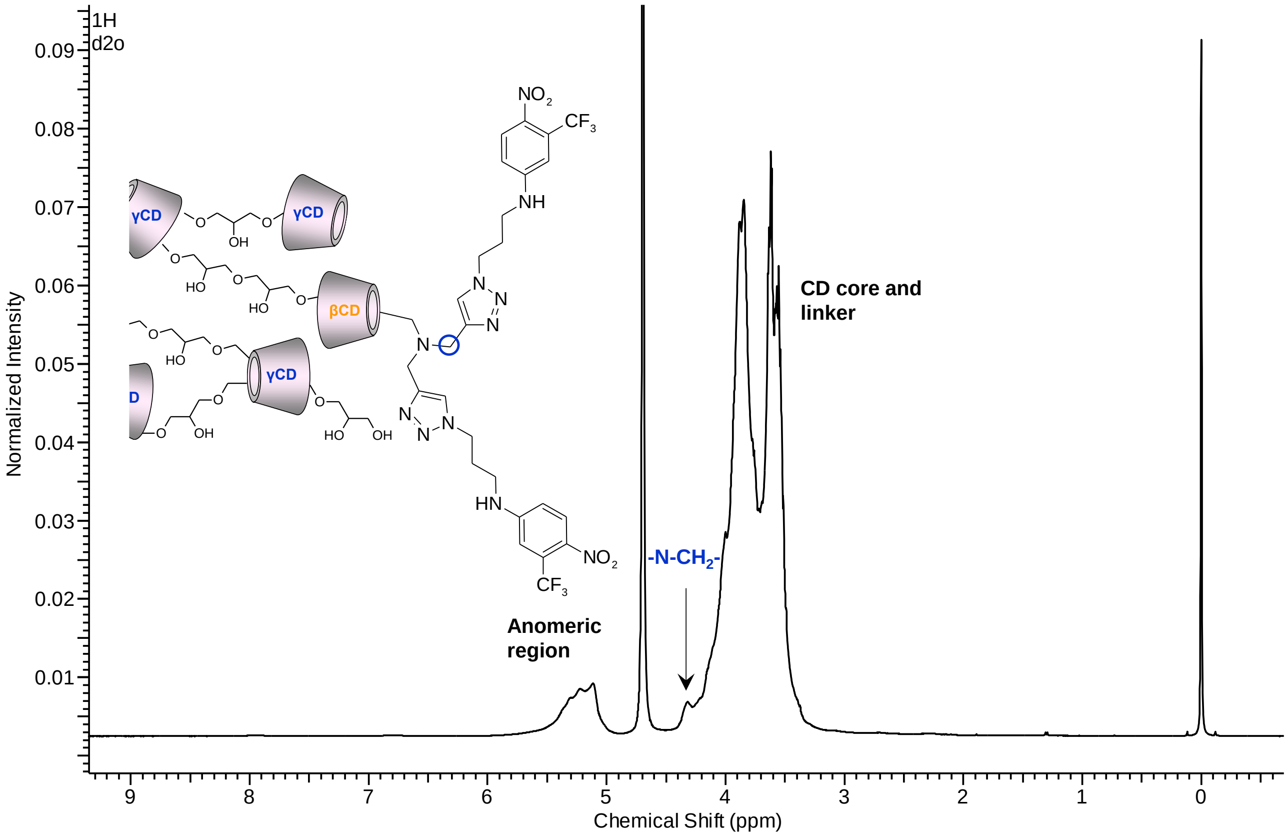

Figure S4. ^{1}H-NMR spectrum of **βγCD-NOPD** with partial assignment (D_2O, 500 MHz, 298 K, with 0.05 wt. % 3-(trimethylsilyl)propionic-2,2,3,3-d_4 acid, sodium salt).

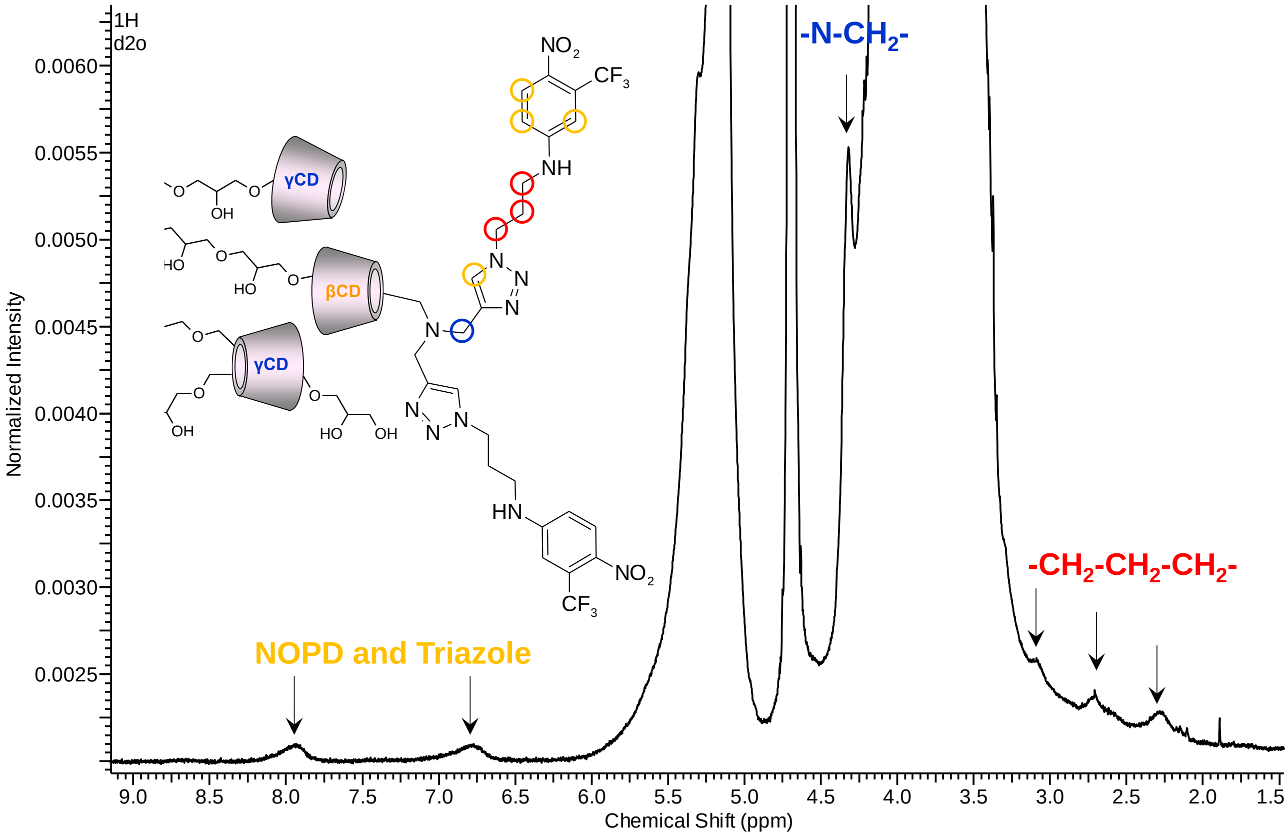

Figure S5. ^{1}H-NMR spectrum enlargement of **βγCD-NOPD** with partial assignment (D_2O, 500 MHz, 298 K, with 0.05 wt. % 3-(trimethylsilyl)propionic-2,2,3,3-d_4 acid, sodium salt).

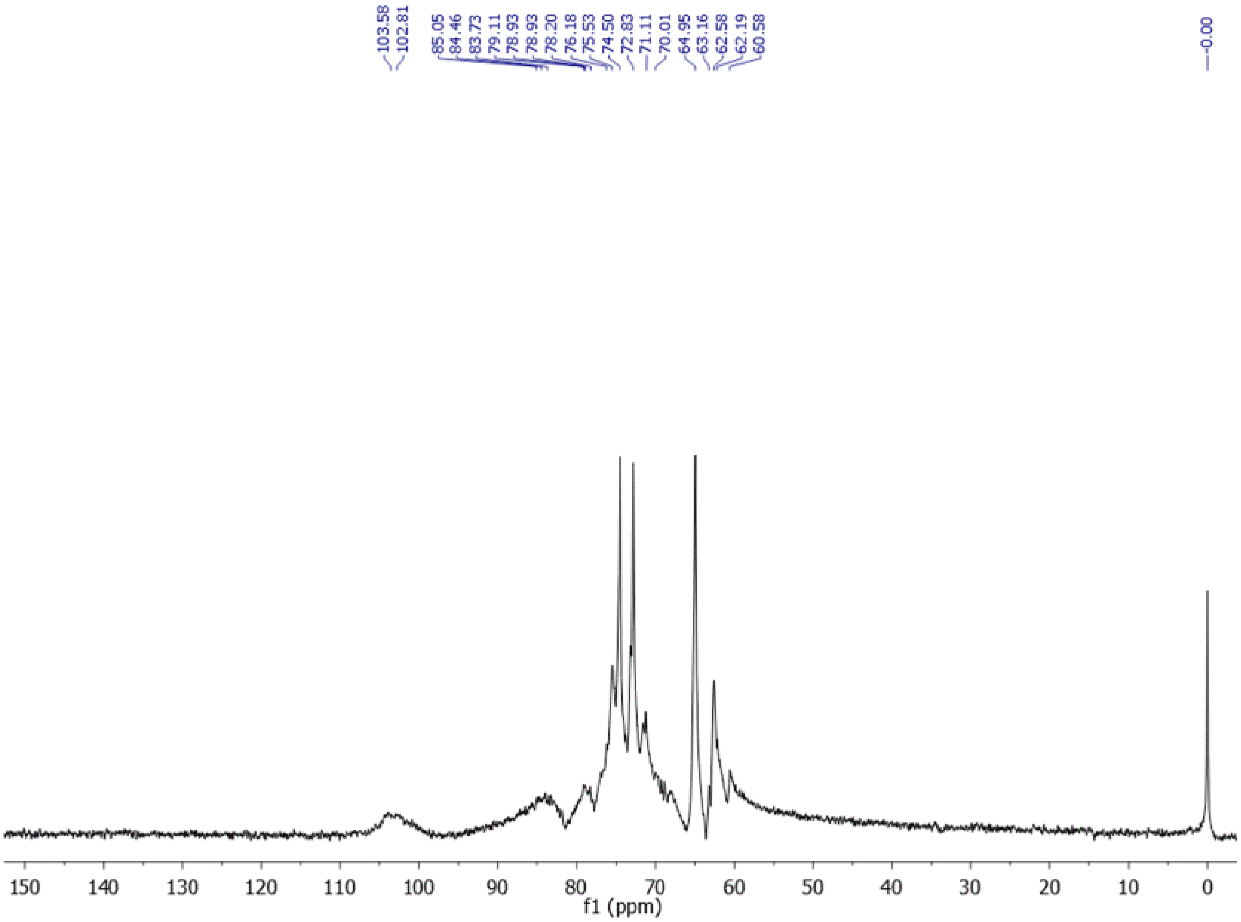

Figure S6. ^{13}C-NMR spectrum of t **βγCD-NOPD** (D_2O, 126 MHz, 298 K, with 0.05 wt. % 3-(trimethylsilyl)propionic-2,2,3,3-d_4 acid, sodium salt).

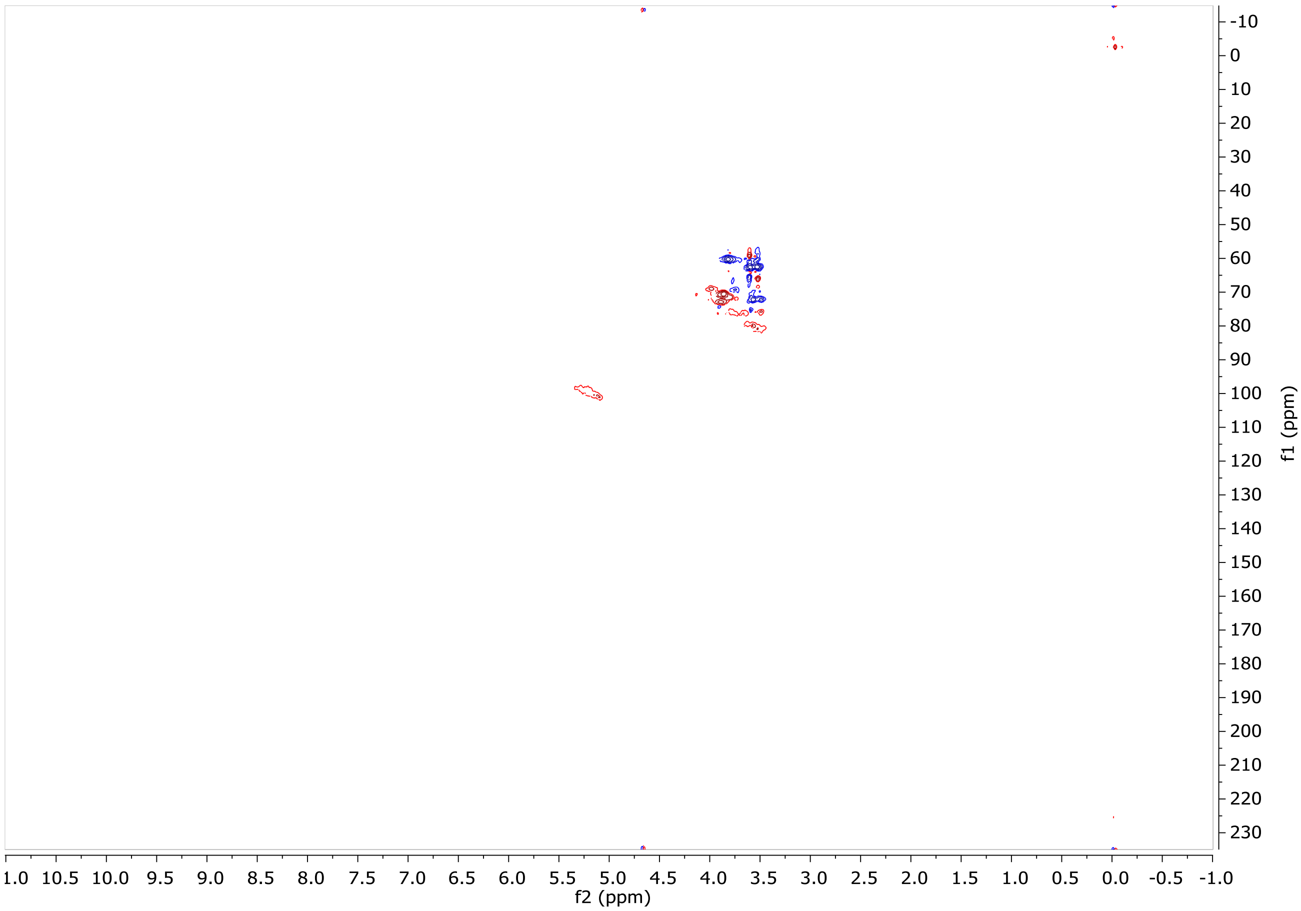

Figure S7. DEPT-edited HSQC spectrum of **βγCD-NOPD** (D_2O, 500 MHz, 298 K, with 0.05 wt. % 3-(trimethylsilyl)propionic-2,2,3,3-d_4 acid, sodium salt).

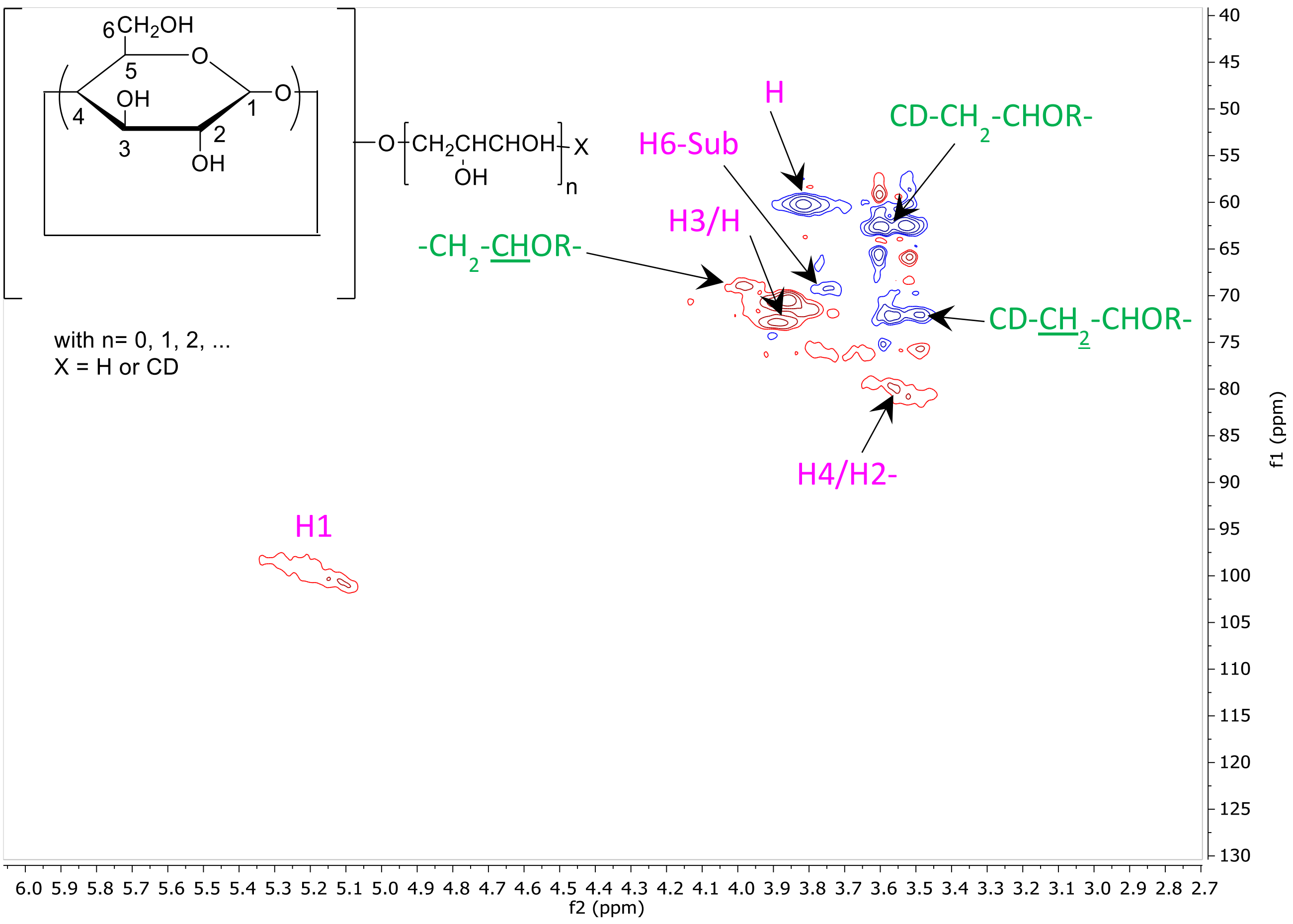

Figure S8. Enlargement of DEPT-edited HSQC spectrum of **βγCD-NOPD** with partial assignment (D_2O, 500 MHz, 298 K, with 0.05 wt. % 3-(trimethylsilyl)propionic-2,2,3,3-d_4 acid, sodium salt.

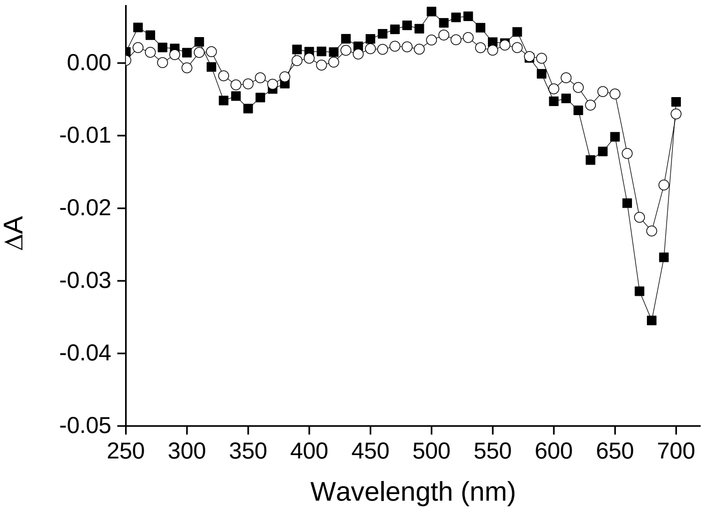

Figure S9. Transient absorption spectra observed 1 µs (■) and 80 µs (○) after 355 nm laser excitation (E_{355} ~ 10 mJ/pulse) of N_2-saturated water solution of **βγCD-NOPD** (2 mg mL^{-1}) loaded **ZnPc** (10 µM).

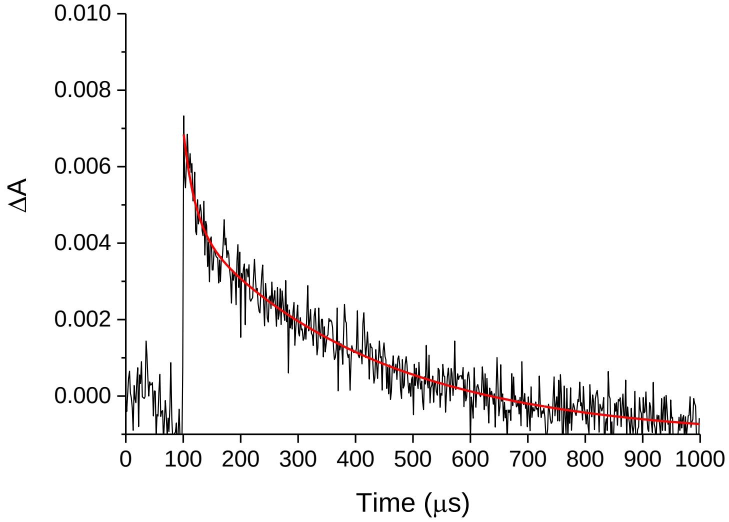

Figure S10. Decay trace and related bi-exponential fitting of the same sample as in Figure S1 monitored at 500 nm under N_2-saturated conditions.

References.

S1. Callari, F. L.; Sortino, S. Amplified nitric oxide photorelease in DNA proximity. *Chem. Commun.* **2008**, *17*, 1971–1973.

S2. Howe, L.; Zhang, J. Z. J. Ultrafast studies of excited-state dynamics of phthalocyanine and zinc phthalocyanine tetrasulfonate in solution. *J. Phys. Chem. A* **1997**, *101 (18)*, 3207–3213.

S3. Tardivo, J. P.; Del Giglio, A.; De Oliveira, C. S.; Gabrielli, D. S.; Junqueira, H. C.; Tada, D. B.; Divinomar S.; de Fátima Turchiello, R.; M. S. Baptista. Methylene blue in photodynamic therapy: From basic mechanisms to clinical applications. *Photodiagnosis Photodyn Ther*. **2005**; *2 (3)*, 175–191.

S4. Puskás, I.; Szemjonov,A.; Fenyvesi, E.; Malanga, M.; Szente, L. Aspects of determining the molecular weight of cyclodextrin polymers and oligomers by static light scattering. *Carbohydrate Polymers*, **2013**, 94, 124-128,
